# Sexual dimorphism in immunity and longevity among the oldest old

**DOI:** 10.3389/fimmu.2025.1525948

**Published:** 2025-02-17

**Authors:** Nelli A. Arakelyan, Daria A. Kupriyanova, Jelena Vasilevska, Evgeny I. Rogaev

**Affiliations:** ^1^ Center for Genetics and Life Science, Sirius University of Science and Technology, Sochi, Russia; ^2^ Department of Psychiatry, University of Massachusetts Medical School, Worcester, MA, United States

**Keywords:** sexual dimorphism, longevity, sex-specific, centenarians, aging, immune response

## Abstract

Human longevity is a sex-biased process in which sex chromosomes and sex-specific immunity may play a crucial role in the health and lifespan disparities between men and women. Generally, women have a higher life expectancy than men, exhibiting lower infection rates for a broad range of pathogens, which results in a higher prevalence of female centenarians compared to males. Investigation of the immunological changes that occur during the process of healthy aging, while taking into account the differences between sexes, can significantly enhance our understanding of the mechanisms that underlie longevity. In this review, we aim to summarize the current knowledge on sexual dimorphism in the human immune system and gut microbiome during aging, with a particular focus on centenarians, based exclusively on human data.

## Introduction

The field of sex-based biology has made significant progress over the last decade, particularly in the areas of healthy aging and lifespan. Extreme longevity, particularly reaching the age of 100 years, is an exceptionally rare trait in the human population, exhibiting significant variations in prevalence between genders. Women generally exhibit greater survival rates than men across all age groups, including centenarians (1, 2), with the gender gap in life expectancy ranging from 4.2 to 6.2 years (3). Genetic factors and immune responses play a crucial role in achieving longevity; however, there is limited information regarding the mechanisms that regulate the differences in biological aging between men and women. From birth, females benefit from genetic advantages in their chromosomal constitution, which is associated with a preponderance of longer telomeres (4). Furthermore, women typically maintain this phenotype better throughout the aging process; however, in the centenarian group, no significant difference in telomere length between men and women was observed (5).

A strong immune system is a key factor in determining lifespan, and sex plays an important role in its composition and activity. In humans, sex is genetically determined by the presence of X and Y chromosomes, with females possessing an XX pair and males having an XY pair. The sex-based diversity in immunity between males and females is regulated by several mechanisms, including X chromosome inactivation, mosaicism, skewing, and dimorphism in the expression of X chromosome-encoded genes, as well as regulatory genes on the Y chromosome (6). Generally, women exhibit stronger innate and adaptive immune responses than men. It is known that young women are more susceptible to autoimmune diseases, while men are more frequently affected by bacterial, parasitic, fungal, and viral infections (7, 8). An additional factor that may contribute to gender differences in the immune system is the gut microbiota. Composition of gut microbiota varies between males and females providing sex-specific differences in immune responses (9). Additionally, various environmental factors may induce different immune responses in males and females, thereby influencing longevity in distinct ways. With age, immune dysregulation manifests differently between the sexes, potentially contributing to the rarity of men surviving to age 100. Although elderly females generally experience poorer health, men of the same age tend to have fewer physical disabilities (10). Numerous studies have reported age-related changes in the composition and functions of immune cells that are sex-specific, as well as differences in secretome production and the activation of signaling pathways (11). However, the majority of such research has been conducted using various animal models, which cannot adequately replicate the human immune microenvironment. Consequently, the data obtained from these studies cannot always be correlated with human responses, leading to a significant lack of human data. Similarly, there is no suitable animal model that can sufficiently represent phenomena observed in human centenarians. In this review, we focus exclusively on human data. Given that sex hormones influence a broad spectrum of biological processes and significantly affect sexual dimorphism in various aspects, we chose not to address this topic in our review. Instead, we opted to reference well-written recent reviews on the subject (12). We discuss the interplay between age and sex in both innate and adaptive immunity, as well as the contribution of sex chromosomes, gut microbiome, and several environmental factors to these effects, with particular emphasis on the limited studies involving centenarians.

## Age-dependent immune cell composition in men and women

It is important to understand and consider the role of gender in the entity of changes that affect the immune system during aging to be able to increase a probability of reaching an advanced age with strong health. In this section of the review, we examine the variations in immune composition between young men and women, as well as the changes that occur during the aging process. The immune system begins to develop very early in intrauterine life, and differences in lymphocyte composition between males and females are present already at that stage. Around two decades ago, the first evidence of sex-based differences in immune system development emerged through studies that tracked male and female cohorts from cord blood at birth up to 18 years of age (13, 14). It was shown that a total lymphocyte number was similar between girls and boys in infancy and was followed by a gradual and significant decline into adulthood. However, the distribution of individual cell subpopulations between genders was slightly different. Thus, girls contained a higher level of CD4+ T cells and higher CD4/CD8 T cell ratios. In contrast, boys exhibited a higher abundance of CD8+ T cells and NK cells. The numbers of B cells and regulatory T (Treg) cells were comparable between both genders of children. Recent studies have provided greater clarity regarding the propagation of specific B cell and T cell subpopulations. Using cord blood as a model, Bous et al. compared sixteen male and twenty-one female newborns. They found that the frequencies of transitional and naïve B cells were similar; however, populations of innate B cells, class switched memory B cells, late memory B cells, plasmablasts, and B2 cells were more prevalent in the cord blood of male newborns. Among T cell populations, only naïve thymus-negative T helper (Th) cells were significantly more abundant in male cord blood, while the other twenty T cell subpopulations showed similar frequencies in both sexes (15).

Throughout development and aging, the composition of immune cells in adults changes, with some immunological differences between genders persisting throughout life, while others only become evident with age-related changes. In the context of healthy aging, centenarians appear to experience a slower rate of aging, making their repertoire of immune cells particularly intriguing. Seminal studies have provided important data on gender- and age-related differences in immune cell populations. For instance, the levels of circulating blood monocytes were found to be higher in young men compared to young women, and these levels were observed to increase in aged individuals and centenarians for both genders (16–20). However, there is evidence that monocytes from females exhibit a more activated phenotype than those from males (21).

The majority of studies discovered a higher prevalence of B cells in women than in men (22–24); however, there is some controversial data in this context (16, 20, 25, 26). In general, an age effect has been observed for both genders, resulting in a slight decrease in B cell numbers (27, 28), with the male cohort showing a greater reduction trend (22–24, 29). Nevertheless, despite the seemingly controversial cell density, the activation of B cells and their transcriptional profiles notably differ between the sexes during aging or in response to pathogens (20, 25).

The density of dendritic cells (DC) has been found to be similar in both young and elderly men and women (16, 20); however, it is reported to be slightly impaired in centenarians (20). While sex-specific phenotypic differences in DC have been observed in *in vitro* studies, they indicate that plasmacytoid DC isolated from females produce higher levels of interferon compared to those isolated from males (30).

A remarkable gender-related increase in the number of natural killer (NK) cells was observed in young men. With aging, the level of NK cells drastically enhanced for both sexes, reaching its peak in centenarians (16, 20, 23, 24, 31). Even though both elderly men and women were able to maintain high levels of NK cells, a greater significance was still noted in men. Notably, women have not only a lower overall number of NK cells, but they also have a relatively smaller percentage of mature NK cells (22).

The activity and distribution of T cell subsets vary significantly between sexes and are known to be differentially affected by aging. Females have higher CD4+ T cell levels and higher CD4/CD8 ratios than males, whereas males have higher CD8+ T cell frequencies (16, 26, 32). Young men exhibited higher levels of regulatory T cells with enhanced immunosuppressive functions compared to women of the same age (33, 34). With aging, the majority of T cell subpopulations decrease in both genders, with a more pronounced reduction observed in men (32). In contrast, the number of Treg cells was found to be enriched in both genders over a lifetime (16, 35, 36). In centenarians, in addition to the ‘younger’ genetic profile of naive T cells (37), a twofold increase in T cells with a cytotoxic phenotype was observed (20), which may ultimately influence longevity. It is also important to note that a decreased level of Treg cells was reported in centenarians compared to an older human cohort (36).

**Figure 1:**
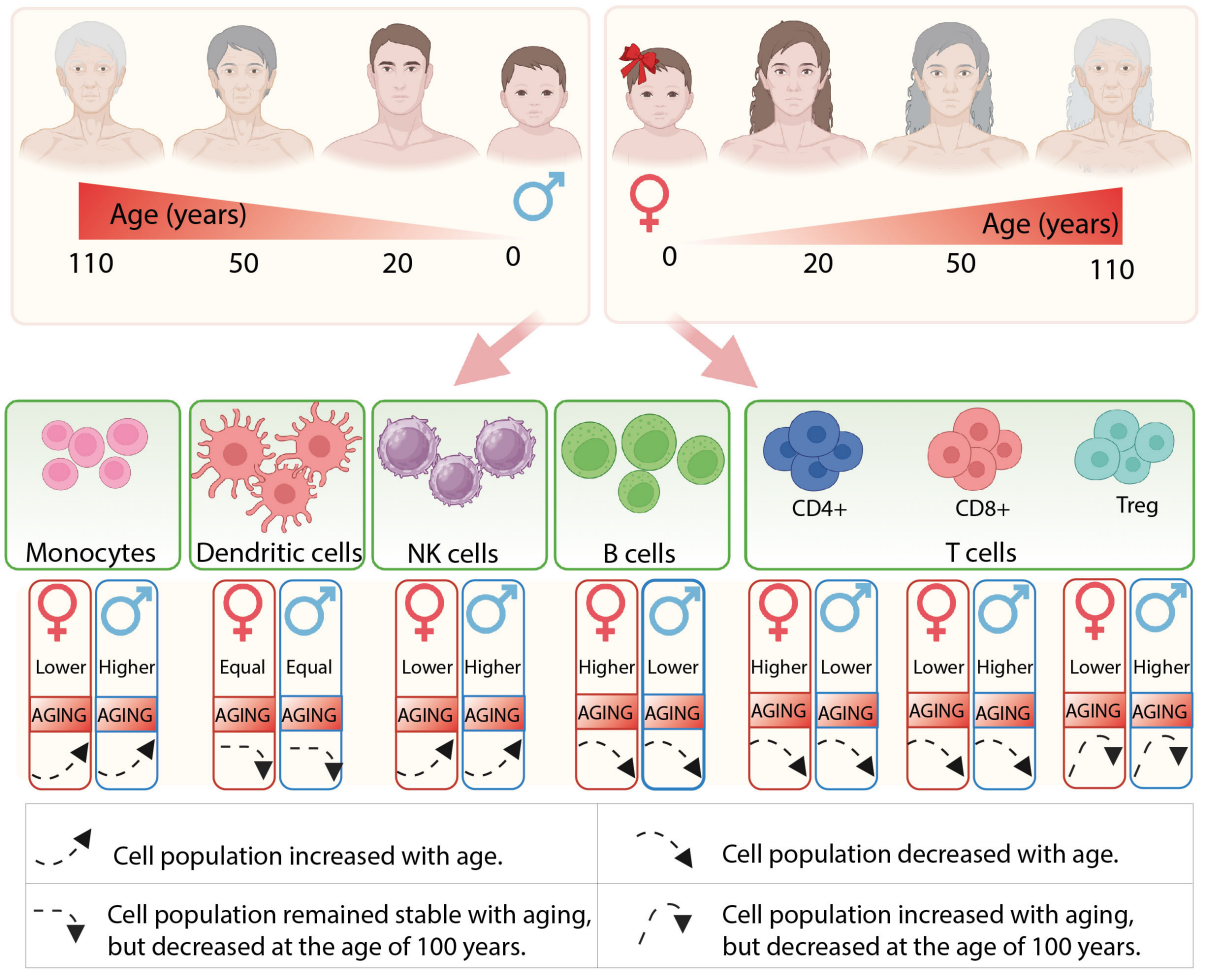
The illustration depicts the composition of immune cells in males and females, as well as the alterations that occur throughout their lifespan.

In summary, there exists considerable variation in the predominance of immune cells between genders, and the composition of immune cells undergoes significant alterations with aging (as summarized in **Figure 1**). Generally, females exhibit stronger innate and adaptive immune responses compared to males. Analyses of the cellular landscape indicate that women demonstrate more active dendritic cells, a higher absolute number of B and CD4+ T cells. Consequently, innate immune cells exhibit more effective phagocytosis and antigen presentation, enhanced production of inflammatory cytokines, and elevated levels of circulating antibodies.

## Immune-related genes regulated by the X chromosome

**Figure 2:**
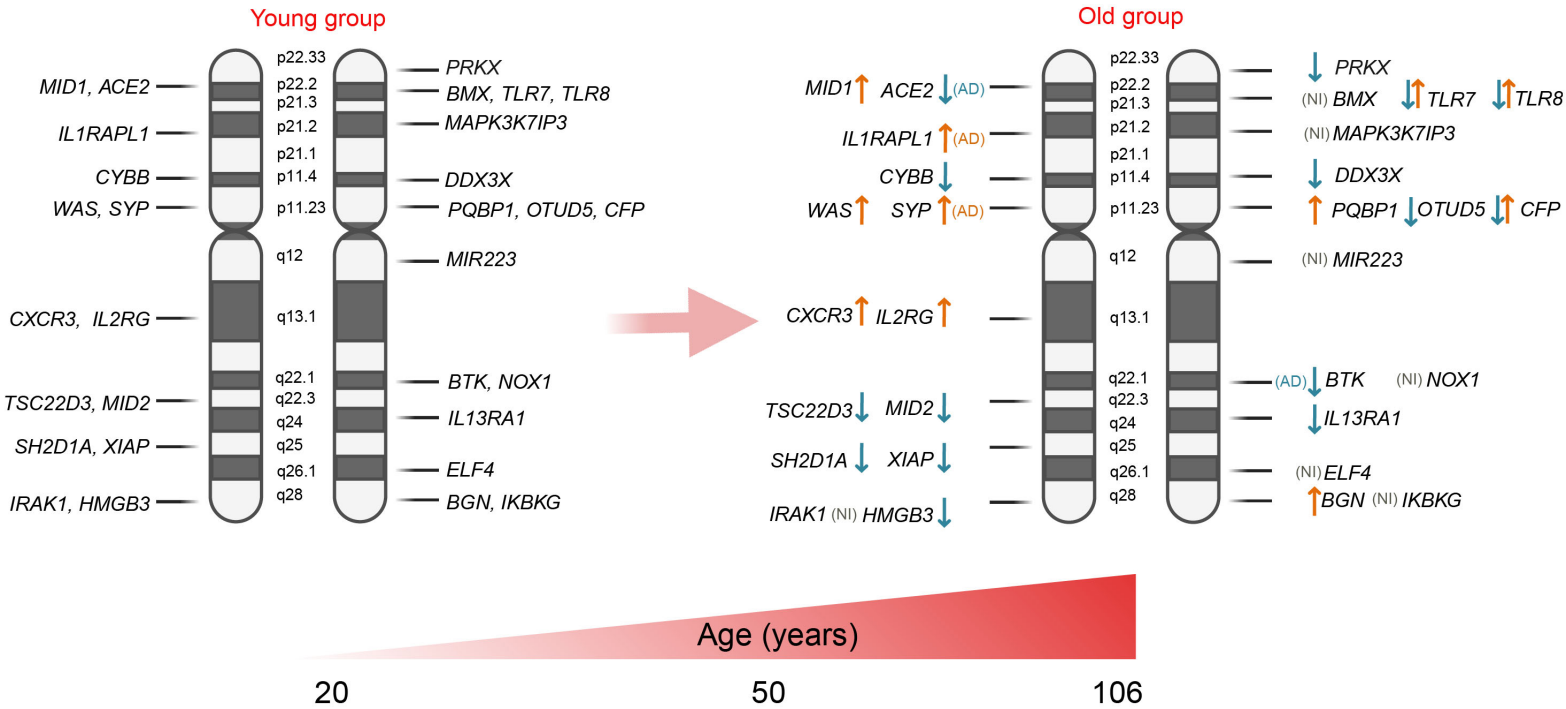
Expression of X-linked genes associated with immunity: ↑ - expression is up-regulated in the older group; ↓ - expression is down-regulated in the older group; and ↑↓ - up- or down-regulated gene expression depending on the cell type. AD, group with Alzheimer’s disease; NI, not identified.

The immune response is modulated by a multitude of factors, with sex chromosomes serving as critical determinants. The X chromosome contains a high density of immune-related genes that play significant roles in both innate and adaptive immune responses. As aging progresses, the composition and activity of immune cells undergo substantial changes, which may be driven by modifications in immunoregulatory gene expression. In this section, we examine the variations in the expression of immuno-associated genes located on the X chromosome throughout the aging process (summarized in **Figure 2** and **Table 1**).

**Table 1:** Expression of immune-associated X-linked genes in young and old groups.

Gene	Cell type/tissue	Technique	Gender	Age of Control group (years)	Age of Case group (years)	Gene expression	Disease	Reference
*DDX3X*	Postmortem frontal cortex	microarray	mix	–	26-106	↓	Healthy	(42)
	memory B cell	scRNA-seq	mix	20-45	60-80	↓	Healthy	(43)
	Dendritic cell	scRNA-seq	mix	20-45	60-80	↓	Healthy	(62)
	Human iPS cell-derived neural progenitor cells	RNA-seq	mix	60-92	60-87 (Sporadic AD)	↓	Alzheimer’s disease	(59)
*TLR7*	Entorhinal cortex Hippocampus Posterior cingulate cortex Superior frontal gyrus	microarray	mix	20-59	60-99	↑ ↑ ↑ ↑(↓)	Healthy	(52)
	Human iPS cell-derived neural progenitor cells	RNA-seq	mix	60-92	60-87 (Sporadic AD)	↓	Alzheimer’s disease	(59)
	Monocyte	scRNA-seq	mix	20-45	60-80	↓	Healthy	(62)
*TLR8*	Entorhinal cortex Hippocampus Posterior cingulate cortex Superior frontal gyrus	microarray	mix	20-59	60-99	↓ ↑ ↑ ↓	Healthy	(63)
	Human iPS cell-derived neural progenitor cells	RNA-seq	mix	60-92	60-87 (Sporadic AD)	↓	Alzheimer’s disease	(59)
	Prefrontal Cortex	RNA-seq	male	46–97 (Age at death)	64–95 (Age at death) Parkinson disease	↓	Parkinson ‘s disease	(64)
*PQBP1*	CD4+ T cell	scRNA-seq	mix	20-45	60-80	↑	Healthy	(62)
	Monocyte		mix			↑	Healthy	(62)
*IL1RAPL1*	Human iPS cell-derived neural progenitor cells	RNA-seq	mix	60-92	60-87 (Sporadic AD)	↑	Alzheimer’s disease	(59)
*PRKX*	CD8+ T cell	scRNA-seq	mix	20-45	60-80	↓	Healthy	(62)
	HMSCs	RNA-seq	male	19–25	75–92	↓	Healthy	(57)
*BTK*	Human iPS cell-derived neural progenitor cells	RNA-seq	mix	60-92	60-87 (Sporadic AD)	↓	Alzheimer’s disease	(59)
*BGN*	Skin fibroblasts	scRNA-seq	female	35-48	70-76	↑	Healthy	(47)
	HMSCs	RNA-seq	male	19–25	75–92	↑	Healthy	(65)
*MID2*	Muscle	microarray	all	18-28	65-84	↓	Healthy	(48)
*MID1*	HMSCs	RNA-seq	male	19–25	75–92	↑	Healthy	(57)
*ACE2*	Human iPS cell-derived neural progenitor cells	RNA-seq	mix	60-92	60-87 (Sporadic AD)	↓	Alzheimer’s disease	(59)
*TSC22D3*	CD8+ T cells	microarray	mix	23-27	65-81	↓	Healthy	(45)
	B cell	scRNA-seq	mix	20-45	60-80	↑	Healthy	(43)
	Monocyte	scRNA-seq	mix	20-45	60-80	↓	Healthy	(43)
	Skin endothelial cell	scRNA-seq	female	18-28	70-76	↓	Healthy	(47)
	Skin fibroblasts	scRNA-seq	female	35-48	70-76	↑	Healthy	(47)
	Human iPS cell-derived neurons	RNA-seq	mix	60-92	60-87 (Sporadic AD)	↑	Alzheimer’s disease	(59)
*WAS*	B cell	scRNA-seq	mix	20-45	60-80	↑	Healthy	(43)
	CD4+ T cell	scRNA-seq	mix	20-45	60-80	↑	Healthy	(43)
	CD8+ T cell	scRNA-seq	mix	20-45	60-80	↑	Healthy	(43)
	Dendritic Cell	scRNA-seq	mix	20-45	60-80	↑	Healthy	(43)
	Monocyte	scRNA-seq	mix	20-45	60-80	↑	Healthy	(43)
	NK cell	scRNA-seq	mix	20-45	60-80	↑	Healthy	(43)
	Human iPS cell-derived neural progenitor cells	RNA-seq	mix	60-92	60-87 (Sporadic AD)	↓	Alzheimer’s disease	(59)
*CXCR3*	CD8+ T cells	microarray	mix	23-27	65-81	↑	Healthy	(45)
	CD4+ T cell	scRNA-seq	mix	20-45	60-80	↑	Healthy	(62)
	CD8+ T cell	scRNA-seq	mix	20-45	60-80	↑	Healthy	(62)
*XIAP*	CD8+ T cell	scRNA-seq	mix	20-45	60-80	↓	Healthy	(62)
*SYP*	Human iPS cell-derived neural progenitor cells	RNA-seq	mix	60-92	60-87 (Sporadic AD)	↑	Alzheimer’s disease	(59)
	Human iPS cell-derived neurons	RNA-seq	mix	60-92	60-87 (Sporadic AD)	↑	Alzheimer’s disease	(59)
*CYBB*	Human iPS cell-derived neural progenitor cells	RNA-seq	mix	60-92	60-87 (Sporadic AD)	↓	Alzheimer’s disease	(59)
	B cell	scRNA-seq	mix	20-45	60-80	↓	Healthy	(43)
	Dendritic Cell	scRNA-seq	mix	20-45	60-80	↓	Healthy	(43)
	Monocyte	scRNA-seq	mix	20-45	60-80	↓	Healthy	(43)
*OTUD5*	HMSCs	RNA-seq	male	19–25	75–92	↓	Healthy	(65)
*IL2RG*	Prefrontal Cortex	RNA-seq	male	46–97 (Age at death)	64–95 (Age at death) Parkinson disease	↑	Parkinson ‘s disease	(64)
	CD4+ T cell	scRNA-seq	mix	20-45	60-80	↑	Healthy	(43)
	CD8+ T cell	scRNA-seq	mix	20-45	60-80	↑	Healthy	(43)
	NK cell	scRNA-seq	mix	20-45	60-80	↑	Healthy	(43)
*CFP*	Skin immune cell	scRNA-seq	female	35-48	70-76	↓	Healthy	(47)
	Monocyte	scRNA-seq	all	20-45	60-80	↑	Healthy	(43)
	Human iPS cell-derived neural progenitor cells	RNA-seq	mix	60-92	60-87 (Sporadic AD)	↓	Alzheimer’s disease	(59)
*IL13RA1*	Monocyte	scRNA-seq	mix	20-45	60-80	↓	Healthy	(43)
	Human iPS cell-derived neural progenitor cells	RNA-seq	mix	60-92	60-87 (Sporadic AD)	↓	Alzheimer’s disease	(59)
	HMSCs	RNA-seq	male	19–25	75–92	↓	Healthy	(57)
*HMGB3*	Human iPS cell-derived neurons	RNA-seq	mix	60-92	60-87 (Sporadic AD)	↑	Alzheimer’s disease	(59)
	HMSCs	RNA-seq	male	19–25	75–92	↑	Healthy	(57)
*SH2D1A*	CD8+ T cells	microarray	mix	23-27	65-81	↓	Healthy	(45)
	CD4+ T cell	scRNA-seq	mix	20-45	60-80	↓	Healthy	(43)

PS, induced pluripotent stem cells; HMSCs, human mesenchymal stem Cells; ↑, expression is upregulated in the case group; ↓, expression is downregulated in the case group; RNA-seq, bulk RNA sequencing; scRNA-seq, single-cell RNA sequencing.

The human X chromosome possesses a unique biology and is present in both males and females. It spans approximately 155 MB in length (38) and contains more than 1000 genes, 897 of which are protein-coding genes, as reviewed by Barbara R. Migeon (39). Due to the fact that women have two X chromosomes while men have only one, the expression of X-linked genes must be equalized between the two sexes. To achieve this, the majority of genes on one of the two X chromosomes in females undergo random inactivation through transcriptional silencing. However, 10-15% of these genes can escape X-inactivation, acting as potential contributors to sexually dimorphic traits, leading not only to phenotypic variability among females but also to clinical abnormalities in patients with an abnormal number of X chromosomes (40). Even though the expression is considered balanced between males and females and detailed comparisons were not conducted in most studies, many genes exhibit sex-biased regulatory targeting patterns, which may lead to different effects.

The inhibitory expression effect was determined for several X-linked genes. One of these is *DDX3X* (DEAD-box helicase 3 X-linked), which plays a role in antiviral activity and the induction of type I interferon (IFN) (41). In 2004, Tao Lu performed a transcriptional profiling study using postmortem samples from the human frontal cortex of 30 individuals aged 26 to 106 years. This study revealed a decrease in *DDX3X* expression with age (42). Supporting these findings, a study conducted by Y. Zheng and colleagues in 2020 demonstrated a significant decrease in *DDX3X* expression in memory B cells from the peripheral blood of older individuals compared to younger individuals (healthy donors). Similarly, *DDX3X* expression was notably reduced in dendritic cells; however, this analysis did not pass multiple comparison corrections (43). These results suggest a potential trend of inhibited *DDX3X* expression in immune cells with age. Expression of transcription factor *TSC22D3* which encodes a glucocorticoid (GC)-induced anti-inflammatory leucine zipper (GILZ) protein and has immunosuppressive functions (44) was detected to be inhibited in CD8+ T cells (45) and monocytes in aged group older than 60 years; however, at the same time, it has risen in B cells (43). Another gene, *SH2D1A*, which is predominantly expressed in T, NK, and some B cells, has been shown to be downregulated in CD4+ and CD8+ T cells with aging. Similarly, the expression of *XIAP* (X-linked inhibitor of apoptosis), which plays a role in both innate and adaptive immunity by modulating TNF-receptor signaling and regulating inflammasome activity, was also reduced in CD8+ T cells of the elderly. The *CYBB* gene, also known as cytochrome b-245 beta chain or *NOX2*, is involved in the production of reactive oxygen species (ROS) by phagocytes. In the older group (ages 60-80), the expression of *CYBB* was detected to be significantly downregulated in B cells, dendritic cells, and monocytes compared to the younger group (ages 20-45) (43). *IL13RA* is also classified as an X-chromosome-regulated gene. *IL13RA1* mediates the JAK1, STAT3, and STAT6 signaling pathways induced by IL-13 and IL-4, which play critical roles in the initiation and regulation of innate immune responses and adaptive immunity (46). Older individuals exhibit a reduction in *IL13RA1* expression in monocytes (43). The *CFP* gene encodes the complement factor properdin involved in regulating the natural immune system’s alternative pathway. It was found to be down-regulated in skin infiltrated immune cells (47), but was detected to be up-regulated in monocytes of the elderly group (43). The expression of *MID2* (midline 2) was found to be decreased in the muscle tissue of elderly individuals (ages 65-84) compared to that of younger individuals (ages 18-28) (48).

*TLR7* and *TLR8* are other important immune-associated genes that are mapped to the X chromosome. As reviewed by Tianhao Duan et al. , toll-like receptors (TLRs) bridge innate and adaptive immunity by influencing antigen-presenting cells and inflammatory cytokines (49). Generally, older adults exhibit dysfunctional activity of TLRs (50), which may contribute to increased infection-related morbidity and mortality, as well as the development of age-associated neurodegenerative diseases (51). Notably, the expression of *TLR7* has been shown to be reduced in monocytes from individuals over 60 years of age (43). Cribbs et al. examined the expression of *TLR7* and *TLR8* genes in various brain regions of young (20-59 years), aged (60–99), and individuals with Alzheimer’s disease (74-95 years), revealing a robust upregulation of these genes in the aged population (52). It is important to note that both *TLR8* and *TLR7* can evade X chromosome inactivation in female immune cells, specifically in monocytes, CD4+ T cells, and dendritic cells, resulting in sex-biased expression. Consequently, the co-expression of genes from both the active and inactive X chromosomes may lead to increased TLR8/TLR7 protein levels in female cells, potentially influencing their response to viruses and bacteria, as well as the risk of developing inflammatory and autoimmune diseases. However, it is also worth mentioning that a similar effect has also been observed in men with Klinefelter syndrome, who possess 47,XXY chromosomes (53–55).

In contrast, the expression of several X-linked genes is activated during the aging process. For instance, the inducible chemokine receptor *CXCR3* has been shown to be upregulated in CD4+ and CD8+ T cells in older individuals (43, 45). Another gene, *WAS*, which encodes the WASP actin nucleation-promoting factor and is a member of the Wiskott-Aldrich syndrome (WAS) family of proteins, is involved in the transduction of signals from receptors on the cell surface to the actin cytoskeleton. With aging, the expression of *WAS* is enhanced in most immune cell populations, namely in B cells, CD4+ and CD8+ T cells, dendritic cells, monocytes, and NK cells (43), however, the data are limited for individuals older than 80-years. *PQBP1* gene encodes polyglutamine binding protein-1, which functions as an intracellular receptor that recognizes pathogens and neurodegenerative proteins (56). This gene was detected to be upregulated in the monocytes and CD4+ T cells of elderly individuals (aged 60-80) compared to younger individuals (aged 20-45) (43). The interleukin 2 receptor subunit gamma (*IL2RG*) serves as an important signaling component for many interleukin receptors and was significantly increased in CD4+ T cells, CD8+ T cells, and NK cells of senior participants (43). Additionally, the expression of other X-chromosome-regulated genes, such as biglycan (*BGN*), was found to be enhanced in the skin fibroblasts of the older group (aged 70-76) compared to the younger group (aged 35-48) (47).

In 2022, Liu et al. conducted a transcriptome analysis of human mesenchymal stem cells (hMSCs) isolated from healthy young males (ages 19–25) and elderly males (ages 75–92) to investigate why younger individuals possess a higher regenerative capacity than their older counterparts. This analysis revealed several X-linked genes that are differentially expressed between the two groups. Specifically, the expression of genes such as *IL13RA1*, *OTUD5* (which regulates interferon signaling), and *PRKX* (involved in monocyte-macrophage function) was inhibited in cells from old individuals. Conversely, the genes *MID1* (Midline-1) and *HMGB3* (high mobility group box protein 3) were found to be overexpressed (57).

There is increasing evidence that X-linked genes influence the aging process and age-related diseases. Meyer et al. generated induced pluripotent stem cell (iPSC)-derived neural progenitor cells and neurons from aged healthy donors (ages 60-92) and aged donors with Alzheimer’s disease (AD) (ages 60-87). The iPSC-derived neural cells from AD patients exhibited accelerated neural differentiation and reduced progenitor cell renewal. These neurons showed upregulation of the X-linked genes *TSC22D3*, *HMGB3*, and *SYP*. Additionally, a similar phenotype was observed in AD iPSC-derived neural progenitor cells, which demonstrated significantly impaired expression of several X-linked genes, including *DDX3X*, *TLR7*, *TLR8*, *BTK*, *ACE2*, *WAS*, *CYBB*, *CFP*, and *IL13RA*. Overexpression was identified solely for the *IL1RAPL1* gene, which is implicated in dendrite differentiation (58) and *SYP* gene which has been shown to be responsible for intellectual disability (59). These findings suggest that the dysregulation of X-linked gene networks may create favorable conditions for the development of Alzheimer’s disease and impact the healthy aging process.

Other X-linked immune-related genes, such as *IRAK1*, *MAPK3K7IP3*, *MIR223*, *NOX1*, *BM*X, and *ELF4*, were not identified as differentially expressed between the young and elderly groups. Research indicates that certain genes, such as *IRAK1* and *MIR223*, play a role in the regulation of inflammatory and infectious responses. These genes exhibit polymorphisms that are associated with sex-specific differences in immune responses. Furthermore, these variations may be influenced by inherited traits and the unique X-chromosome mosaicism present in females, which contributes to the regulation of immune responses as aging (59–61).

## Y chromosome in immunity and longevity

The role of the Y chromosome in human diseases has been overlooked for many years, and a great paradigm shift regarding its biological importance has only occurred recently. There are a limited number of human studies that have examined the impact of the Y chromosome on lifespan. In 2023, Teoli et al. demonstrated transcriptome changes in men with different karyotypes (46,XX, 46,XY, 47,XXY and 47,XYY) showing that the number of Y chromosomes negatively affects longevity. The authors claimed that an additional Y chromosome leads to increased expression of transposable elements (TEs), thereby disrupting genomic stability. Overall, the results obtained in this study support the hypothesis regarding the toxicity of the Y chromosome (66).

The length of the Y chromosome is significantly shorter than the X chromosome, estimated to be approximately 62 MB. The Y chromosome contains 693 genes, of which 106 are protein-coding (67). Genes linked to the Y chromosome are called “Holandric”, and in contrast to X-linked genes, Y-linked genes do not directly regulate either innate or adaptive immunity, but they may indirectly influence immune system functions. In 2009, Sezgin et al. studied 3,727 males in a cohort of HIV-positive patients to investigate whether major Y haplogroups are associated with HIV infection and progression. They genotyped 11 common Y haplogroups in European Americans and African Americans and found that American men with haplogroup I lineage exhibited faster disease progression and a higher mortality rate at seven years post-infection. Additionally, individuals with the Y-I haplogroup tended to experience more rapid T cell depletion, which resulted in delayed responses to viral load suppression following antiretroviral therapy (68). These results suggest that the Y chromosome is implicated in the regulation of T cells.

The Y chromosome is essential for male health, and mosaic loss of chromosome Y (mLOY) has been shown to have a strong correlation with aging. Namely, mLOY has been observed to increase with advancing age, with detection rates of 40% in males aged 70 years and 57% in males aged 93 years and among centenarians (69–72). Therefore, mLOY is considered a potential marker of biological age and is associated with certain age-related diseases, such as neurodegenerative disorders (73), cardiovascular diseases (74), macular degeneration (75), and Alzheimer’s disease (76). Furthermore, in aging men, mLOY has been linked to cancer with an advanced aggressive phenotype (77, 78).

The Y chromosome is known not to code for immune-related genes; therefore, mLOY serves as a suitable model to study the indirect effects of the Y chromosome on immunity. In 2019, Thompson et al. addressed the question of whether the loss of a Y chromosome from circulating leukocytes has a direct functional effect and whether loss of Y chromosome in leukocytes serves as an indicator of broader genomic instability. A study was conducted involving the analysis of 205,011 men aged between 40 and 70 years, which revealed that hematopoietic stem and progenitor cells exhibited the highest enrichment for cell types associated with variants linked to LOY. These observations suggest that LOY may have a direct impact on hematopoietic stem cells rather than on more differentiated white blood cell types. Additionally, by examining PBMC from nine male donors aged 64 to 89 at a single-cell resolution, they demonstrated dysregulation of the *TCL1A* gene in B-lymphocytes with LOY (70). Notably, expression of *TCL1A* in T and B cells was shown to disrupt cycle transitions and hinder adequate DNA damage response, leading to genomic instability (79). This provides proof of the concept that LOY may be functionally active in these cells. Other evidence suggesting that the Y chromosome may be implicated in immune regulation was published by Dumanski et al. (80). By analyzing PBMCs from men aged 64 to 94 with prostate cancer or Alzheimer’s disease, they demonstrated that LOY is associated with dysregulation of autosomal gene expression in leukocytes. It is important to note that LOY exhibited cellular specificity; patients with Alzheimer’s disease were preferentially affected by LOY in NK cells, whereas in patients with prostate cancer, LOY was observed in CD4+ T cells and granulocytes.

## Sexual dimorphism in response to environmental factors and immunity

**Figure 3:**
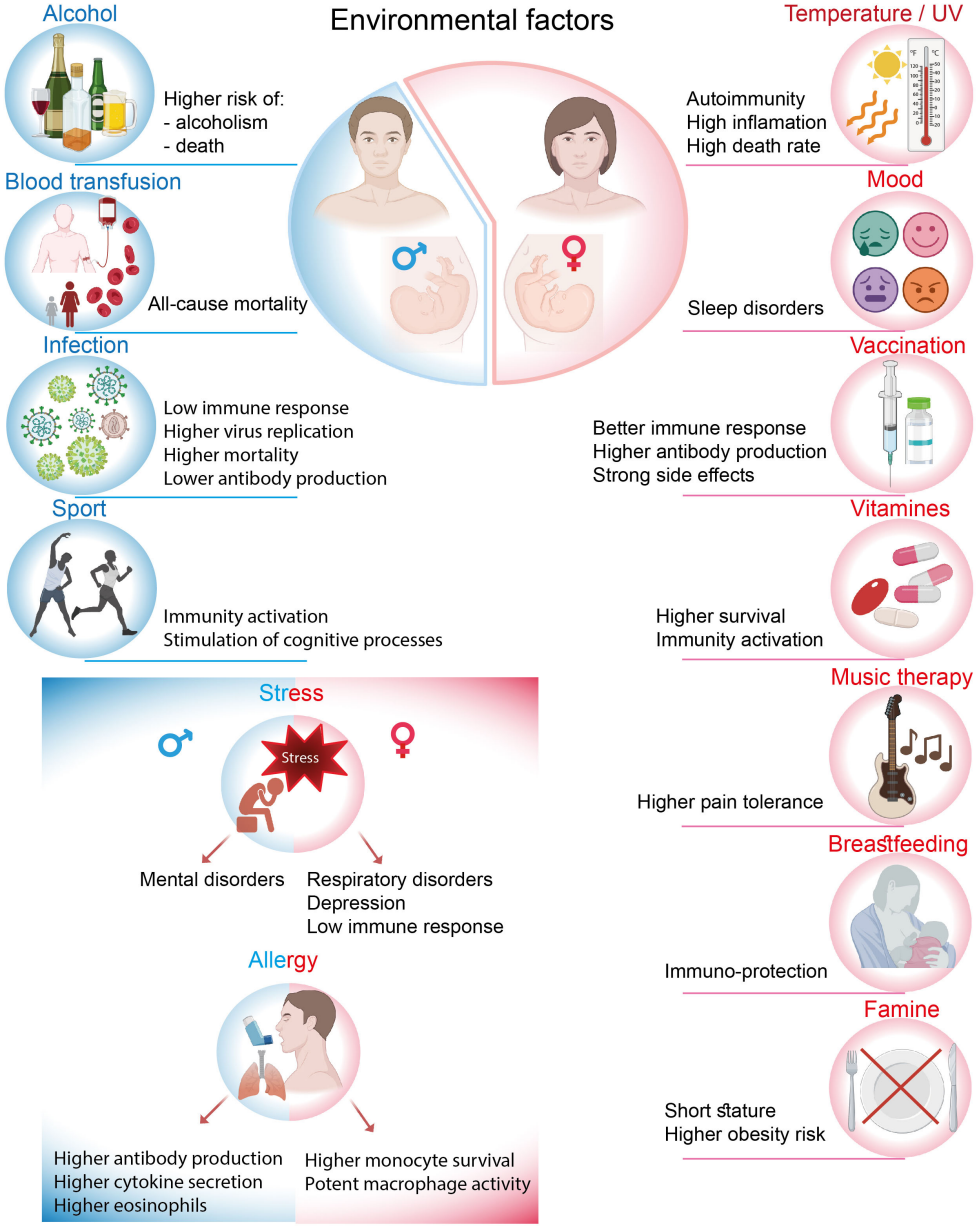
Different effects of environmental factors on men and women.

The immune system is activated not only in response to pathogens but also in reaction to environmental exposures, where sexual dimorphism is also observed. The concept of external influences is multifaceted and is recognized by the immune system from early life. This section discusses how various environmental factors, including chemical, biological, physical, and physiological influences, can affect immune activity in both men and women (summarized in **Figure 3**).

### Habitat/climate

The human habitat, which encompasses various factors including the level of development of the country or weather fluctuations such as precipitation and sunny days, may influence immune responses and the composition of immune cells. For instance, there are grounds to declare that the levels of T-lymphocyte subsets in healthy children from developed countries are lower compared to those in Guinean children, who exhibit markedly higher total lymphocyte percentages but decreased CD4+ T cell percentages and CD4+/CD8+ T cell ratios. Furthermore, with respect to this topic, it was observed that during the rainy season, the percentages of lymphocytes and the CD4+/CD8+ T cell ratio were significantly lower in comparison to the dry season; however, the levels of CD8+ T cells exhibited an increase (13).

Men and women may experience divergent immune response trajectories due to biological, environmental, and social factors, with temperature being one of the sex-biased influences. In 2022, when Europe experienced heatwaves with record temperatures, 56% more heat-related deaths were recorded in women than in men, likely due to a combination of biologic and societal factors (81). At the core, there are physiological differences between the sexes that begin with body temperature and heat response. On average, females are 0.1 to 0.5°C warmer than males, with more pronounced fluctuations in their extremities. In this context, sexual dimorphism is notably observed in the susceptibility to rheumatoid arthritis among women. Elevated temperatures in a joint may preferentially activate Th17 T cells, which subsequently generate additional heat and secrete inflammatory cytokines, thereby intensifying the inflammatory process (82, 83). However, body temperatures decrease with age in both sexes (84, 85). Furthermore, sex also influences the effects of UV radiation on autoimmune disorders. Research has shown that UV radiation may modulate the clinical and immunological expression of autoimmune diseases predominantly in women, while significant associations were not detected in men (86).

### Stress and mood disorders

An environmental factor is a broad concept that encompasses several aspects. Maternal stress is one of the most potent external factors that can differentially affect male and female embryo development during pregnancy. Research has shown that maternal life stress during pregnancy or postnatally is associated with an increased risk of adverse respiratory outcomes in childhood, particularly in girls (87). Conversely, boys appear to be more vulnerable to developing mental disorders, such as schizophrenia, under similar conditions (88). Stress can be detrimental not only on its own but can also contribute to depression. Among adults, women have been observed to be more susceptible to stress and mood disorders, with a prevalence ratio of 2:1 between women and men (89, 90). The investigation of immune biomarkers in males and females diagnosed with depression revealed that both sexes exhibited elevated white blood cell counts. However, men demonstrated a greater increase in leukocyte levels, which is a hallmark of depression, compared to women (91). The lymphocyte and neutrophil chemotaxis index was also significantly higher, indicating enhanced migratory ability. Other immune processes, such as neutrophil phagocytosis, lymphocyte proliferation, and NK cell activity, did not show any gender-related differences. Another symptom associated with mood disorder is insomnia, which is more prevalent in women. For instance, in response to the COVID-19 pandemic, women reported a longer time to fall asleep, an increased frequency of awakenings during the night, lower sleep efficiency, and poorer sleep quality compared to men (92). However, the immunomodulatory mechanisms underlying the progression of insomnia remain unknown. Sexual dimorphism also occurs in response to intriguing factors such as art therapy, which is regarded as a mood-driven body-mind treatment. For example, music therapy has been used as a complementary therapy for cancer patients (93), and its effects on pain tolerance and pain ratings were examined using the cold pressor test. Ghaffaripour et al demonstrated that music therapy was more effective for women, claiming that they are more easily influenced and distracted from pain than men. Importantly, maximal pain tolerance was significantly enhanced when participants listened to their preferred music, compared to Iranian folkloric music or no music at all (94). This indicates that music can serve as a powerful emotional stimulus, but only when there is an emotional connection with a particular listener.

### Diseases and treatments

Males and females differ in the intensity, prevalence, and pathogenesis of viral infections. In particular, females are more shielded and the responses to viruses are usually more intense than in males. For instance, females exhibit higher antiviral responses to HIV, as HIV-infected women show a greater number of CD4+ T cells, increased activation levels of CD8+ T cells, and enhanced plasmacytoid dendritic cell (pDC) activity. In untreated HIV-infected children, females were found to have lower HIV RNA levels than males, indicating inhibited viral replication (95, 96). Additionally, there are gender differences in immune reconstitution among HIV patients receiving antiretroviral therapy. Women achieve a faster and more robust long-term immune response, demonstrating sufficient levels of CD4+ T cells, which are associated with lower risks of disease-induced morbidity and mortality (97). However, it is also important to note that men tend to exhibit a more casual attitude toward treatment, resulting in more frequent interruptions in therapy compared to women (98).

In the context of SARS-CoV-2 virus infection, sexual dimorphism begins at the embryonic stage, where gender-related differences have been identified in the placental expression of the *TMPRSS2* gene. This gene is one of the host molecules essential for the entry of SARS-CoV-2. *TMPRSS2* expression was found to be significantly higher in male placentas compared to female placentas, indicating a sex-specific vulnerability to placental SARS-CoV-2 infection (99). In adults, males accounted for 59–68% of infection cases and exhibited higher mortality rates (100). This disparity can be attributed to the fact that during SARS-CoV-2 infection, women demonstrated more robust T cell activation, while men exhibited higher levels of cytokine secretion, such as IL-8 and IL-18, along with a more pronounced induction of non-classical monocytes (101). In contrast to anti-HIV therapy, vaccination against COVID-19 was reported to have lower acceptance rates among women compared to men (102). However, at the same time, post-vaccination side effects from COVID-19 were observed to be more prevalent in females than in males (103).

Females tend to exhibit more symptoms of influenza. They also develop stronger humoral immune responses to influenza vaccines and experience more adverse side effects post-vaccination, while producing higher levels of antibodies than males (104–106). Similar to COVID-19, immunization rates against influenza are lower in women, which may be attributed to greater perceived vaccination-associated risks. In contrast, males exhibit a higher susceptibility to tuberculosis and develop a weaker immune response following vaccination. *Ex vivo* experiments with human PBMC demonstrated that vaccinated females exhibited a higher frequency of cytokine-producing NK cells and a higher CD4/CD8 ratio. In males, there was a trend toward a higher IFN-γ response and an increased frequency of monocytes (107). Additionally, it was demonstrated that the production of HBeAg and HBsAg antibodies was higher in the serum of female HBV patients compared to that of male patients (108).

In childhood, boys have a higher risk of developing allergic reactions; however, during adolescence, this tendency shifts in favor of women, particularly concerning asthma and food allergies (109) Generally, allergic asthma is triggered by enhanced type 2 inflammation, which includes elevated levels of cytokines such as IL-4, IL-5, and IL-13, as well as increased production of IgE antibodies. An analysis of allergic asthma progression among children, where boys exhibited greater vulnerability, indicated sex-specific differences in immune responses. This was evidenced by increased secretion of IFN-gamma, IL-5, and IL-13, along with higher total IgE levels and elevated peripheral eosinophil counts in males (110). In adults, where women predominate, the effects of the IL-4-mediated signaling pathway in monocyte-derived macrophages in the lungs were shown to be sex-specific. First, monocytes from asthmatic women exhibited higher *CX3CR1* expression, which enhances macrophage survival. Second, these monocytes demonstrated a greater capacity to adopt a more potent M2 macrophage phenotype in response to IL-4 polarization, a factor that is crucial for responsiveness to allergic asthma (110).

Blood transfusions, a crucial medical procedure, have been shown to induce sex-biased outcomes. Several studies have demonstrated that transfusions from donors to recipients of different sexes can lead to varying consequences, which are not observed when both the donor and recipient are of the same gender (111). Particularly noteworthy is the finding that recipients who received blood transfusions from ever-pregnant female donors experienced a statistically significant increase in all-cause mortality, but this effect was observed only among male recipients, not females. This correlation was not detected in men who received transfusions from female donors without a history of pregnancy (112). This phenomenon can be explained by a hypothesis suggesting that women develop a long-term immunological memory of pregnancy. The establishment of this immunological memory may be considered an evolutionary advantage for women, essential for succeeding in future pregnancies and ensuring maternal well-being. However, this adaptation appears to be poorly compatible with the male organism. Generally, pregnancy induces modifications in immune cell subsets, driven by maternal methylation of specific genes that regulate the differentiation and function of T cells and NK cells. Namely, pregnancy affects the proportions of T cells, including T helper 2, cytotoxic T cells, and homing memory CD8+ T as well as ratios of immature/mature NK cells, leading to a significant decrease in these populations. Levels of immunological background in postpartum women remained stable for a minimum of one year (113). Although the modifications to the immune system that occur in women during and after pregnancy have been described, the underlying mechanisms that negatively affect men following blood transfusion remain elusive.

### Food

Food is another environmental factor that can affect individuals in a gender-dependent manner. For instance, Wang et al. (2010) analyzed the potential association between famine and pregnancies during that period and its impact on adult body weight. They reported that the famine in Chongqing from 1959 to 1961 predominantly affected female infants, leading to shorter adult height and higher rates of obesity, while the impact on boys was less pronounced (114). Additionally, the effect of vitamin supplements on children born by HIV-infected women was found to be sex-specific. The beneficial effects on the risk of low birth weight and reduced mortality were observed mainly among girls, with no significant effects noted for boys (115). Similarly, vitamin A, which serves as a supplement during vaccinations, promoted an increase in the numbers of leukocytes, lymphocytes, monocytes, and basophils in female children and was also associated with enhanced IFN-γ responses to phytohaemagglutinin (116). Clearly, the biological mechanisms underlying these observations warrant further investigation; however, it is speculated that the outcomes may be influenced by sex-related differences in immunological factors.

It is well known that breastfeeding offers numerous health benefits for infants, providing essential nutrients and immune protection. Research has shown that the gender of the infant can influence the biochemical composition and energy content of a mother’s breast milk. Mothers of male infants tend to produce milk with higher levels of protein and mature fat compared to those with female infants. Conversely, mothers of female infants have been found to have higher concentrations of carbohydrates, glucose, and lactose in their colostrum. As a result, male infants consume milk with a higher energy density (117, 118). In 2003, Sinha et al.investigated the impact of breastfeeding on community-acquired neonatal infections in a study cohort of 13,224 mother-infant pairs. Interestingly, only female infants, not males, were found to be protected from neonatal respiratory tract infections through breastfeeding. These findings suggest that the immunomodulatory effects of breast milk may also be sex-biased (119). Additionally, the lower rates of breastfeeding among female infants corresponded with increased mortality rates, indicating that the lack of immunoprotection provided by breast milk heightened female infants’ vulnerability to waterborne diseases (120).

Alcohol consumption is a food-related topic that is often characterized as a male-dominated activity leading to subsequent alcohol-related injuries. Men are at a significantly higher risk of developing alcohol addiction; however, women face a greater risk of organ damage and other medical consequences associated with alcohol consumption (121). Nevertheless, alcohol use disorder is the second most disabling disease and injury condition affecting men (122). Generally, alcohol has a suppressive effect on immunity. There is evidence that alcohol consumption induces a sex-dependent immunomodulatory effect. Long-term alcohol consumption of 0.33 ml bear resulted in an increased population of CD3+ T cells only in women, whereas 0.66 ml had no effect on men. As a consequence, alcohol stimulated immune response by amplifying antibodies IgG, IgM, IgA and cytokines IL-2, IL-4, IL-10, and IFN-gamma production exclusively in women (123).

### Sport

Sexual dimorphism also pertains to the outcomes of physical activity. Casaletto et al. investigated whether there are differences in physical activity levels and systemic inflammatory and brain health outcomes between older men and women. Interestingly, a significant beneficial effect of daily physical activity was observed only in men, not in women. They discovered lower concentrations of MCP-4, MCP-1, MDC, MIP-1β, INF-γ, and eotaxin-3, as well as larger parahippocampal volumes and improved visual memory and processing speed, none of which were observed in women. These findings suggest that immune activation driven by physical exercise may be male-specific and could impact brain health (124).

In summary, susceptibility to environmental factors in men and women is a complex and multifaceted phenomenon that results in different modulations of the immune system and wellbeing outcomes between genders. The findings indicate that women are more frequently influenced by environmental factors, both positively and negatively.

## Centenarians: women versus men

Given the diverse nature of the approaches and findings in the field of longevity, the following sections will primarily focus on the sexual dimorphism observed in centenarians. We will highlight the broad heterogeneity of factors that may influence centenarians lifespan, including genetic, physical, economic, social, and cultural aspects, as well as lifestyle choices.

**Figure 4:**
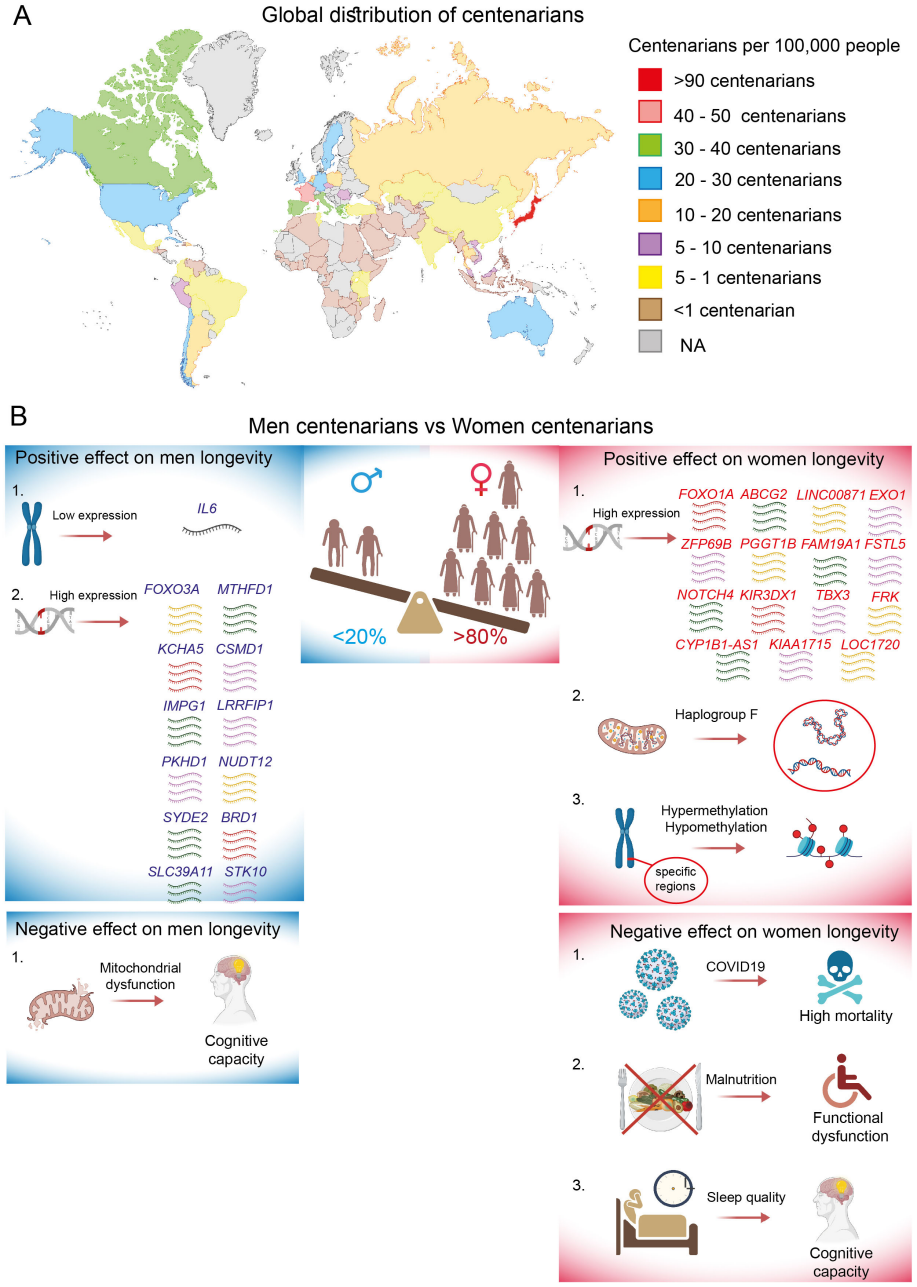
Global distribution of centenarians and sex-specific longevity effects. **(A)** The density is represented as the number of centenarians per 100,000 people for the year 2024, specifically in countries with populations exceeding 10 million inhabitants. Data is marked as NA (not analyzed) for countries with populations below 10 million inhabitants. **(B)** Sex-specific positive and negative effects on centenarian longevity mediated by genetic and environmental factors.

The prevalence of centenarians has increased globally over the past few decades, reaching approximately 600,000 individuals by 2024 (125). In 2016, Buettner et al. found that a larger number of centenarians was associated with specific geographical areas known as blue zones, where they proposed to discover the healthiest lifestyles that contribute to vitality and longevity (126). However, the distribution of centenarians in 2024 (**Figure 4A**) clearly indicates that the density of the world’s longest-lived individuals also strongly correlates with the development status of the country and cultural patterns. Life expectancy is greatly higher in developed countries compared to developing countries, where advancements in preventive healthcare, treatments, social care, and overall quality of life are much more advanced. Japan stands out as the country with the highest number of centenarians, boasting approximately 97 individuals per 100,000 people. This phenomenon can be attributed not only to the unique Japanese diet and lifestyle but also, and more importantly, to economic success and robust health insurance systems that led to notably reduced mortality rates from ischemic heart disease and cancer (127).

France is a second leading country in terms of longevity, often referred to as the “French paradox”. It is the concept that highlights the significant role of moderate wine consumption in promoting longevity, particularly through its positive therapeutic effects on ischemic heart disease, metabolic syndrome, cognitive decline, depression, and cancer (128). Next, countries such as Canada, Italy, Greece, Spain, and Portugal boast more than 30 centenarians per 100,000 inhabitants. Following closely are countries like the US, Australia, Sweden, Germany, England, Belgium, Chile, and Cuba, which have more than 20 centenarians per 100,000 people. Conversely, countries with the fewest super-agers are primarily located on the African continent. Africa faces serious health challenges, characterized by a wide range of diseases and limited access to essential medicines (129). As a result, African countries have conducted little or no research on centenarians. Therefore, when summarizing the geographical distribution of the world’s extreme long-livers, it becomes evident that one substantial factor contributing to longevity may be the balance between a particular lifestyle and quality of life.

In general, more than eighty percent of centenarians are women, despite the paradoxical fact that men tend to have better health at this age (130). This observation of the gender paradox remains difficult to explain. The prevalence of females among centenarians has been observed in all countries, with the exception of Mexico, where the number of centenarians slightly favors men over women. Intriguingly, in 2017, the ratio of male to female centenarians was radically different, indicating that there were three times as many women as men. However, it has been reported that in Mexico, females face more unfavorable conditions compared to men, particularly in terms of loneliness, limitations in physical and social activities, and health issues (131).

### Genetic factors

More than 25% of the variability in human lifespan is estimated to be genetic (132); however, it seems unlikely that this genetic phenotype is solely hereditary (133). In the last decade, several studies have identified numerous genes and genetic loci that promote longevity, including *APOE*, *TOMM40*, *HLA-DQA/DRB1*, *LPA*, *SHC1*, and insulin/IGF1 signaling, which are considered sex-independent genetic risk factors (134–136). However, the genetic contribution to longevity traits has been shown to be moderated by gender, with females exhibiting a stronger genetic background. Additionally, genetic influence appears to be highly specific to ethnicity. Thus, the analysis of non-synonymous SNPs in DNA repair genes among German and French centenarians revealed a significant enrichment of the functionally relevant SNP rs1776180 in female centenarians. This SNP was associated with increased *EXO1* expression, positioning it as a novel candidate gene for longevity in women (137). In the same context, a sex-specific genome-wide association study (GWAS) and polygenic risk score analysis were conducted using a cohort of Chinese centenarians to identify longevity loci specific to males and females. The longevity-associated genes that were most significant for women from the northern and southern regions of China included *ZFP69B*, *PGGT1B*, *FAM19A1*, *FSTL5*, *NOTCH4*, *KIR3DX1*, *CYP1B1-AS1*, *TBX3*, *KIAA1715*, *FRK*, and *LOC1720*. Notably, a deeper comparative analysis of the Chinese women-specific loci with available datasets revealed only two longevity-associated genes that were identified to be common between Chinese and U.S. women, as well as between Chinese and European women: *LINC00871* and *ABCG2*, respectively (138). In another study, *FOXO1A* was detected to be more strongly associated with longevity in Chinese females than in males (139).

In contrast, specific genes associated with longevity in Chinese male centenarians include *MTHFD1*, *KCHA5*, *CSMD1*, *IMPG1*, *LRRFIP1*, *PKHD1*, *NUDT12*, *SYDE2*, *BRD1*, *SLC39A11*, and *STK10* (138). However, unlike the cohort of women, no common gene associations have been published between Chinese and European or U.S. men. Additionally, there is evidence that inhibited IL6 gene expression is significantly associated with longevity in Italian male centenarians (140). Controversial data have been published regarding the *FOXO3A* gene. Several studies indicate that *FOXO3A* is associated with longevity in both sexes among individuals of German, Japanese, French, and Chinese descent. However, some data suggest a predominant association of *FOXO3A* specifically with the lifespan of Italian men (139, 141–143). An analysis of sex-specific pathways involved in the regulation of human lifespan reveals a significant activation of metabolic pathways in female centenarians, while immune- and inflammatory-related pathways are predominantly activated in male centenarians (138).

Polymorphisms in the coding regions of mitochondrial DNA play a pivotal role in human longevity across various ethnicities, exhibiting a sex-unbiased effect (144–146). However, some sex-specific genetic influences have also been reported. Notably, mitochondrial haplogroup F has been significantly associated with exceptional longevity exclusively in Chinese females. The authors suggested that haplogroup F is linked to higher levels of HDL and a functional SNP variant (m.13928G>C) (147).

It appears that ethnicity significantly influences variations in results and should be taken into account, particularly in the investigation of genetic factors, which is currently under intense scrutiny. For example, genes such as *TOMM40*, *APOC1*, and *APOE*, which have previously been shown to correlate significantly with longevity in European women, have not been replicated in studies involving African American and Hispanic women. This discrepancy is likely due to the fact that different ethnicities may exhibit a higher prevalence of certain alleles compared to others (148).

### Epigenetic factors

DNA methylation represents a crucial epigenetic modification that facilitates the interaction between genetic and environmental factors, and it has demonstrated potential in elucidating the mechanisms that contribute to exceptional healthy longevity. The epigenetic clock, which relies on DNA methylation patterns, is widely recognized and utilized to estimate chronological age or to identify biological age. In recent study, the epigenetic clock was calibrated using data from 336 Japanese participants. An analysis of the epigenetic ages of Japanese centenarians and supercentenarians revealed that all participants exhibited younger epigenetic ages compared to their chronological ages, accompanied by negative epigenetic-age acceleration (149). Similar findings were observed in a cohort of French centenarians (150). These results imply that healthy longevity may possess an epigenetic basis. However, no evidence of sexual dimorphism was detected in either study. In contrast, the epigenetic age predictors for the old oldest men from Italy and Greece demonstrated lower correlations with chronological age and exhibited positive epigenetic age acceleration when compared to women from the same regions (151). Additionally, a study conducted by Sun et al. provided a comprehensive comparison of the DNA methylome between the longest-lived men and women in the Chinese population. Although the average methylation levels of the examined CpGs were comparable, women exhibited DNA hypermethylation and hypomethylation in specific chromosomal regions and genes, particularly on chromosome 17, which harbors numerous disease-associated genes (152).

### Other factors

During the COVID-19 pandemic, older individuals were more susceptible to infection and had faced the highest risk of death. Due to the differing responses to infections between the two genders described above, older females exhibit greater resilience to COVID-19 than males. However, in the context of centenarians, women have a higher mortality rate, which may be attributed to immune changes resulting from lifelong stress (153). In general, there is statistical evidence that centenarians born before 1919 were more resilient to COVID-19 compared to other elderly individuals. This phenomenon has been speculatively attributed to a specific immune response developed following the Spanish flu epidemic of 1918 (154, 155).

Long noncoding RNAs (lncRNAs) serve as critical regulatory elements and have been shown to significantly impact various biological processes as well as the mechanisms associated with human diseases. However, the roles of lncRNAs in the aging process and their correlation with sex remain largely underexplored. In the study conducted by Jiang et al., the authors investigated centenarian-specific lncRNAs within a cohort of long-lived individuals from both the Northern and Southern regions of Hainan Province in China. The study identified 11 lncRNAs that were overexpressed in centenarians compared to control cohorts, with 8 of these lncRNAs being proposed as candidates for promoting healthy aging and longevity. Nonetheless, the study did not address the issue of sexual dimorphism in this context (156).

Cognition and physical capacity are essential functions that serve as important indicators of healthy aging. Consequently, age-related cognitive and physical changes in centenarians have been examined from various perspectives. For instance, mitochondrial dysfunction associated with oxidative stress and, consequently, age-related neurodegeneration was investigated from a gender perspective. Mitochondrial functions such as basal respiration, ATP production, reserve capacity, and maximal respiration were assessed in PBMC from Danish centenarians. A significant inverse correlation between cognitive performance and mitochondrial function was observed only in male centenarians, suggesting a stronger relationship between cognitive ability and mitochondrial functional capacity in men (157). In contrast, cognitive impairments such as difficulties with orientation, calculation, and memory in centenarian women were associated with poor sleep quality and shorter nighttime sleep. One potential explanation for this observation may be that women generally have a higher susceptibility to sleep disorders, and the consequences of this factor are more pronounced in female centenarians than in their male counterparts (158).

Physical independence is another age-related indicator in which women experience greater challenges than men. For instance, poor nutritional status has been linked to increased functional limitations among Chinese female centenarians (159), while men were not significantly affected. Additionally, low levels of total cholesterol have a mediating effect on body mass index and blood pressure. This factor was strongly correlated with a higher frequency of disabilities exclusively among Chinese female centenarians (160).

Psychology is an additional discipline that can significantly contribute to longevity by emphasizing the importance of mental health, which may differ between the sexes. Generally, among centenarians, anxiety, low self-efficacy, and a lack of optimism have been shown to correlate with poorer subjective health and feelings of desperation (161). Building on this concept, personality, character, and temperament profiles were compared between Greek male and female centenarians. Female centenarians exhibited higher levels of adaptability and were more reward-dependent, while male centenarians displayed a more optimistic attitude. From a sociodemographic perspective, men were less likely to report experiences of widowhood and more likely to mention having first-degree relatives who are centenarians, as well as engaging in negative habits such as smoking (162).

In conclusion, human longevity is a multifaceted trait influenced significantly by both environmental factors and genetic predispositions. The investigation of genetic mechanisms that regulate human longevity has the potential to influence healthy aging by delaying or preventing age-related diseases. However, it is evident that favorable genotypes are not consistently replicated across populations and cannot fully account for the gender longevity gap. Therefore, additional factors that influence human lifespan, such as environmental stimuli, lifestyle, and immunity, must also be considered (summarized in **Figure 4B**). In general, the basic biological mechanisms associated with favorable biological signatures for extreme longevity are still in their early stages of understanding. To enhance the healthy lifespan of the increasing population of older adults, further research on the health status of centenarians should be conducted, with a focus on large-scale studies across different countries. These studies should take into account various genetic backgrounds, ethnicities, and cultural traits.

## Sexual dimorphisms in microbiome-regulated disorders

**Figure 5:**
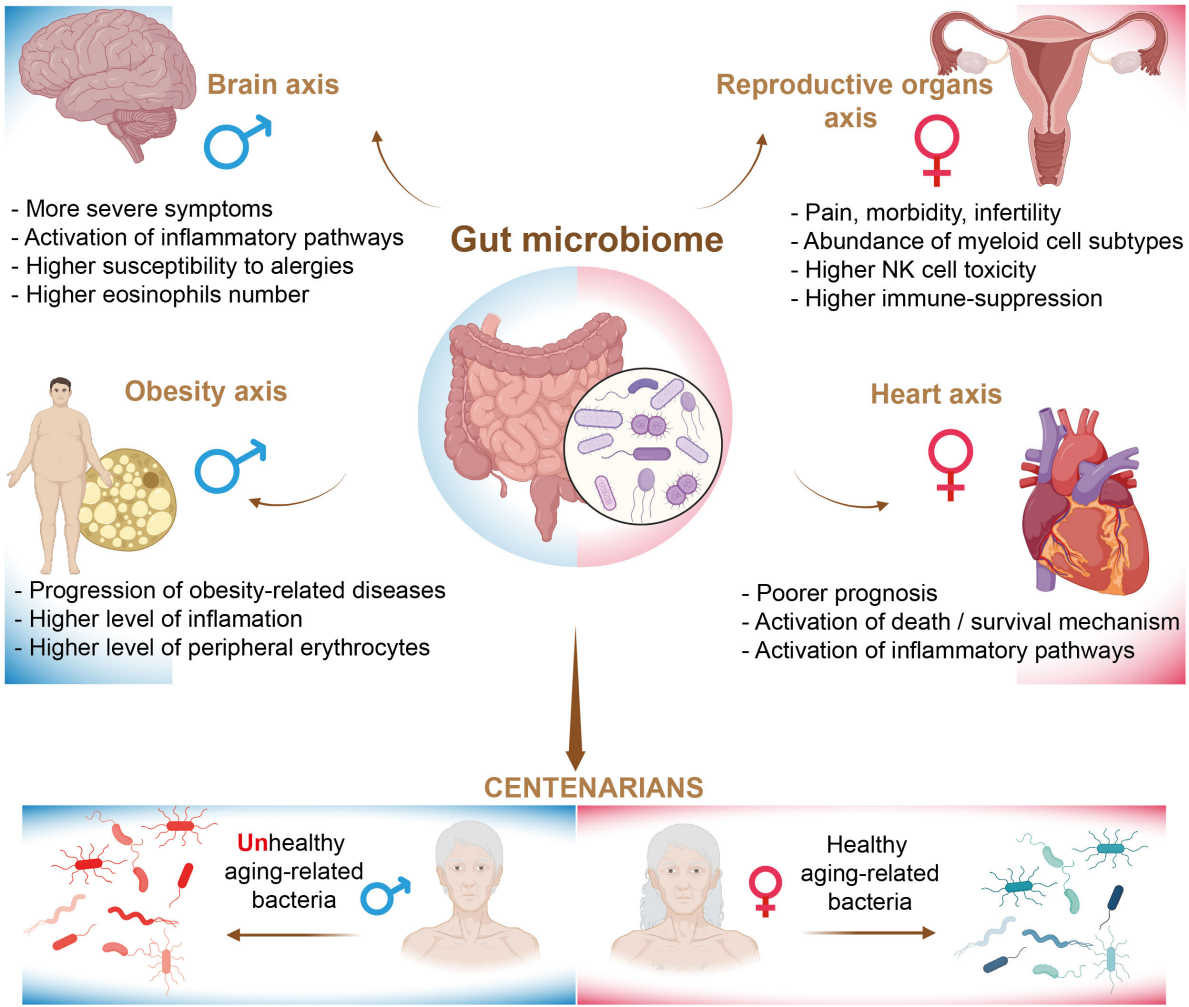
The regulation of health disorders by the gut microbiome.

The gut microbiome plays a crucial role in the development and education of the human immune system. Research indicates that men and women exhibit sex-specific differences in both their immune systems and the composition of their gut microbiota, which may contribute to the observed disparities in immunity and health disorders between the sexes. The microbiome landscape undergoes significant changes throughout the human lifespan, a phenomenon extensively described by Valeri et al. in their review (163). Notably, it has been demonstrated that the ratio of bacterial cells to human cells differs between males and females, with a ratio of approximately 1.3:1 for males and 2.2:1 for females (164). It is well established that factors such as gender, ethnicity, lifestyle, and various environmental influences can significantly affect the status and composition of the gut microbiota (165). Consequently, delineating the functional configurations of the gut microbiome in relation to specific health disorders in humans presents significant challenges. As a result, the majority of studies have primarily utilized germ-free rodent models, thereby underscoring a notable deficiency in clinical research involving humans. In this section, we examine sexual dimorphism in the gut microbiome and the associated immune-related alterations in relation to various health disorders in humans (**Figure 5**). However, we have chosen not to address autoimmune disorders in our analysis, as recent reviews have extensively covered the topic of sexual dimorphism in this context (166–168).

### Gut-brain axis dysfunction

In contemporary research, the investigation of the human microbiome, particularly with respect to the microbiome-gut-brain axis, has catalyzed a significant paradigm shift in the fields of neuroscience and mental health. This research elucidates the existing disparities between men and women in the prevalence of brain- and behavior-related disorders (169, 170). The gut and brain interact in a bidirectional manner through immune, endocrine, and neural pathways. It has been proposed that the differential development of various neurological and psychiatric disorders, such as autism, anxiety, and schizophrenia, is associated with distinct immune responses in men and women (171).

#### Autism spectrum disorder

Autism spectrum disorder is characterized by deficits in social communication and interaction, with the microbiome playing a significant role in its etiology (172, 173). The prevalence of ASD is notably higher in males, with a ratio of approximately 3-4:1 in favor of males (174). Furthermore, the symptoms and progression of ASD often differ between genders, particularly concerning intellectual functioning. Research indicates that females tend to be less severely affected in the domains of communication and repetitive stereotyped behaviors compared to their male counterparts (175). Autistic girls have demonstrated social motivation and friendship quality comparable to that of neurotypical girls, whereas autistic boys have reported qualitatively different friendships and reduced motivation for social interaction (176).

There is evidence indicating that hypovitaminosis may influence the pathophysiology of ASD by modulating the gut microbiota-immune-brain axis (177). Additionally, studies have shown that individuals with ASD exhibit a higher abundance of *Proteobacteria* compared to their healthy counterparts. In contrast, the abundance of *Bifidobacterium*, *Blautia*, *Dialister*, *Prevotella*, *Veillonella*, and *Turicibacter* has been found to be reduced in autistic children, while *Lactobacillus* and *Clostridium* were increased. However, the impact of gender on the microbiome associated with ASD has been overlooked in previous research (178). A comparative analysis of the inflammatory secretome between male and female patients diagnosed with ASD did not indicate a statistically significant difference. It is noteworthy that only one chemokine, macrophage migration inhibitory factor (*MIF*), demonstrated a variation in female patients, with levels elevated in the ASD cohort relative to healthy controls; however, this effect was not evident in male patients (179). Furthermore, in the context of allergy susceptibility, boys with ASD exhibited a markedly higher propensity for respiratory and skin allergies in comparison to their female counterparts. However, no significant differences between sexes were observed concerning food allergies (180).

#### Schizophrenia

Schizophrenia is a mental disorder that is characterized by disruptions in thought, cognitive perceptions, responsiveness, and social interactions. The progression of the disease manifests differently in men and women. Males exhibit a higher prevalence, greater severity, and an earlier age of onset of the condition. Schizophrenic men tend to display a greater prevalence of negative symptoms, which include social withdrawal, blunted affect, poverty of speech, and amotivation. Conversely, research indicates that schizophrenia in women is often characterized by a higher incidence of affective and paranoid symptoms. These symptoms may include dysphoria, depression, irritability, hostility, inappropriate affect, impulsivity, sexually inappropriate or bizarre behavior, and sexual delusions (181). Disruptions in the gut microbiome have been reported to influence the progression of schizophrenia-spectrum disorders. Generally, schizophrenia is characterized by an increased abundance of *Anaerococcus* and decreased levels of *Haemophilus*, *Sutterella*, and *Clostridium* family bacteria. Furthermore, a higher severity of depressive symptoms was positively correlated with an increased abundance of the genus *Bacteroides* and reduced abundance of *Ruminococcaceae* (182).

There is a notable lack of knowledge concerning sexual dimorphism in the gut microbiota associated with schizophrenia. In the study conducted by Severance et al., the authors investigated the effects of lifetime exposure to the opportunistic pathogen *Candida albicans* and observed that males with schizophrenia exhibited a higher level of exposure to this pathogen. Conversely, women diagnosed with schizophrenia and infected with *C. albicans* demonstrated poorer cognitive functions, particularly showing declines in memory module performance, compared to their counterparts who tested negative for *C. albicans* (183). These findings may suggest that *C. albicans* influences the development of schizophrenia in a sex-specific manner.

Numerous studies indicate significant alterations in the immune system associated with schizophrenia, characterized by an increased number of monocytes, B cells, and neutrophils (184–186). However, a sex-specific comparison of immune cell composition in individuals with schizophrenia was conducted over 40 years ago, revealing a statistically significant enrichment of eosinophils in males (187). A recent study conducted by Lobentanzer et al in 2019 employed a comprehensive approach that integrated large-scale transcriptomic meta-analysis of brain tissues from patients with single-cell sequencing data of central nervous system (CNS) neurons, short RNA sequencing of cell lines derived from both male and female humans, as well as connectomic analyses of transcription factor and microRNA interactions with perturbed transcripts. The findings revealed a significant divergence between the sexes among individuals diagnosed with schizophrenia, resulting in a complete absence of overlap in schizophrenia-related genes between male and female patients. Further analysis highlighted an elevation of inflammation- and immunity-related genes in males, while more specific immune processes were identified in females, particularly concerning individual mechanistic components such as interleukin-6 (188). It is evident that further research examining sex differences in the microbiome in conjunction with immune responses is crucial.

### Gut-heart axis dysfunction

Cardiovascular disease continues to be a predominant cause of mortality on a global scale. Cardiovascular diseases are often perceived as primarily affecting men; however, women typically experience the onset of coronary heart disease approximately ten years later than men. Furthermore, women may be as much as twenty years older than their male counterparts when they encounter cardiac events, such as myocardial infarction. Nevertheless, the prognosis following a myocardial infarction is poorer for women in comparison to men (189). Furthermore, women are at an elevated risk of developing Takotsubo cardiomyopathy (190). Additionally, diabetes increases the risk of cardiovascular disease by three to seven times in women and by two to three times in men (191). Recent studies have indicated that individuals with cardiovascular disease exhibit a distinct composition of gut microbiota compared to healthy individuals, with this variation often correlating with inflammation and disease progression. Specifically, a higher cardiovascular risk has been positively associated with an increased abundance of *Collinsella stercoris*, *Flavonifractor plautii*, and *Ruthenibacterium lactatiformans*, while a decrease in the abundance of *Streptococcus thermophilus* has been observed (192).

#### Stroke

The majority of published data regarding stroke do not explicitly differentiate between sexes. Nevertheless, women experience a disproportionately higher burden of stroke-related mortality and disability (193). However, sex-specific microbiota signatures have been identified in patients with ischemic stroke. Research indicates that levels of bacteria belonging to the phylum *Fusobacteria*, class *Fusobacteriia*, order *Fusobacteriales*, and family *Fusobacteriaceae* are elevated in women compared to men (194). It is noteworthy that there is a limited number of studies that have directly demonstrated sex-specific immune alterations associated with cardiovascular diseases. For example, in the context of conditions that may increase the risk of ischemic stroke, research has indicated that in women, the prevention of ischemic stroke correlates with a higher count of reticulocytes. Conversely, in men, a greater incidence of stroke is linked to an elevated coefficient of variation in the complexity of lymphocyte intracellular structures (195). A sexually dimorphic profile of immune cell expression was observed following cardioembolic stroke. Gene expression analyses were conducted on blood samples collected three hours post-stroke. The results indicated a greater number of upregulated genes compared to downregulated genes in both sexes; however, women exhibited a higher number of differentially expressed genes than men. These differentially expressed genes were associated with processes related to cell death and survival, cell-cell signaling, and inflammation. Notably, the pathways identified included toll-like receptor signaling, PPARα/RXRα activation, hypoxia signaling in the cardiovascular system, mitochondrial dysfunction, renin-angiotensin signaling, and glucocorticoid receptor signaling, as well as signaling pathways involving TREM1, IL-10, CD28, IL-1, EGF, HMGB1, FGF, and IL-15. In contrast, no canonical pathways were identified in males that met the false discovery rate corrected P-value threshold. Alterations in gene expression in males were observed only 24 hours after the stroke and the initiation of treatment, which were associated with cellular assembly, organization, and compromise (196).

#### Hypertension

Hypertension affects both men and women; however, the prevalence of hypertension varies between the sexes throughout the lifespan. In early adulthood, men exhibit a higher prevalence of hypertension compared to age-matched women. This trend continues until approximately the sixth decade of life, at which point the prevalence of hypertension becomes either equal to or greater in women (197). Certain bacterial species have been demonstrated to have a strong association with hypertension in patients of both sexes. These species include *Anaerovorax*, *Butyricicoccus*, *Vampirovibrio*, *Methanobrevibacter*, *Oxalobacter*, *Cellulosibacter*, *Mogibacterium, Sporobacter*, *Alistipes finegoldii*, and *Eubacterium siraeum* (198, 199). In a sex-specific context, *Ruminococcus gnavus*, *Clostridium bolteae*, and *Bacteroides ovatus* exhibited significantly higher abundance in hypertensive women, whereas *Dorea formicigenerans* was found to be more prevalent in normotensive women. Notably, no bacterial species demonstrated a significant association with hypertension in men (200). Hypertension is generally correlated with elevated production of interleukin-1 and interleukin-6, as well as increased circulating levels of interleukin-1 receptor antagonists. Additionally, there is a higher number of circulating CD4+ and CD8+/CD45RO+ T cells that produce higher amounts of interleukin-17A compared to controls (201). However, no sex-specific investigations regarding the immune response to hypertension have been conducted in human subjects.

#### Coronary heart disease

A notable sexual dimorphism is observed in the incidence and prevalence of coronary artery disease, with men being more frequently affected than age-matched women. Research has demonstrated that women diagnosed with CHD display a higher prevalence of the *Actinobacteriota phylum*, particularly the *Coriobacteriia class*, along with the *Bifidobacteriales*, *Barnesiellaceae*, and *Tannerellaceae* families. Furthermore, the genera *Parabacteroides*, *Ruminococcaceae incertae sedis*, and *Bilophila* are also found to be more abundant in the gut microbiota of these females. In contrast, the gut microbiota of males is characterized by a predominance of the *Clostridia* family, in addition to the *Prevotellaceae* and *Erysipelotrichaceae* families, as well as the *Eubacterium siraeum* group and the genera *Lachnospira* and *Roseburia* (202). The proportion of immune-related cells, including activated dendritic cells, NK cells, central memory CD8+ T cells, immature B cells, mast cells, monocytes, neutrophils, regulatory T cells, T follicular helper cells, type 17 T helper cells, activated CD4+ T cells, activated CD8+ T cells, central memory CD4+ T cells, effector memory CD8+ T cells, immature dendritic cells, myeloid-derived suppressor cells (MDSC), memory B cells, natural killer T cells, plasmacytoid dendritic cells, and type 2 T helper cells, were found to be significantly reduced in patients with CHD when compared to the control group (203). Furthermore, there is a lack of published data regarding sex-specific differences in the immune context.

### Gut-reproductive organs axis dysfunction

The gut microbiome is recognized as an endocrine organ capable of influencing distant organs and their associated biological pathways. Recent advancements in research indicate that maintaining gut microbial homeostasis is crucial for reproductive health, and those disruptions in the gut microbiota may contribute to reproductive pathologies.

#### Endometriosis

Among the most significant findings from recent studies is the strong association between gut microbiota and endometriosis. Endometriosis is characterized by the growth of uterine-like tissue outside the uterus and is estimated to affect approximately 10% to 15% of women of reproductive age worldwide, leading to considerable pain, morbidity, and infertility (204). Endometriosis is an estrogen-dependent disease; however, its etiology remains largely unknown. Nevertheless, both genetic and environmental factors have been shown to significantly contribute to the risk of developing this condition. Recent studies have indicated that alterations in gut microbiota, particularly an increase in the *Erysipelotrichia* class, were associated with progression (205). Furthermore, the bacterial phyla *Actinobacteria*, *Firmicutes*, *Proteobacteria*, and *Verrucomicrobia* were found to be significantly more abundant in the gut microbiota of individuals with endometriosis compared to the control group. Conversely, the family Lactobacillaceae exhibited a significant reduction in abundance. Additionally, elevated levels of *Proteobacteria*, *Enterobacteriaceae*, *Streptococcus*, and *Escherichia coli* were observed across multiple microbiome sites in the endometriosis cohort (206). Other studies have demonstrated the predominance of *Shigella* in the gut microbiota of women diagnosed with endometriosis (207). The mechanisms through which the gut microbiota contributes to endometriosis are multifaceted, particularly involving immune inflammation. Recent studies have demonstrated the involvement of specific immune cell populations in disease progression. For example, Shin et al. applied single-cell RNA sequencing technology to analyze the immune landscape in endometrial cysts, superficial peritoneal endometriosis, and deep infiltrating endometriosis in female patients. In general, the findings indicate that various cell types including endometrial, lymphoid, macrophage, fibroblast, and endothelial cells exhibit alterations in the context of endometriosis. Notably, within the immune-related framework, an increase in monocyte-derived macrophages was observed across all analyzed tissues. Additionally, an enrichment of tissue-specific myeloid cell subtypes was identified; for example, FCGR3A/CD16+ non-classical monocytes were found to be elevated in cases of deep infiltrating endometriosis, while resident monocytes were enhanced in ovarian endometrial cysts and superficial peritoneal endometriosis (208).

There is substantial evidence indicating that NK cells play multifaceted roles in the progression of endometriosis. A study conducted by Shin et al. demonstrated that NK cells exhibited elevated levels of cytotoxicity markers, while natural killer T (NKT) cells, which co-express T cell and NK cell markers, showed overexpression of checkpoint genes. This finding suggests processes of immune activation and immune cell exhaustion. However, conflicting data exist indicating that NK cell activity and cytotoxicity may be inhibited in women with endometriosis, with this inhibition correlating with the severity of the disease (208). In these cases, NK cells displayed an upregulation of killer inhibitory receptors (*KIR*) and alterations in antigenicity due to the overexpression of human leukocyte antigen (*HLA*) class molecules (209, 210). Changes in the activity of dendritic cells have also been observed. Suszczyk et al. demonstrated that the accumulation of Gal-9-expressing dendritic cells in peripheral blood may serve as a hallmark of immune regulation in patients with endometriosis, potentially exacerbating the inflammatory process and contributing to the development of local immunosuppression (211). In addition, the proportion of T cells was altered, with a notable reduction in CD8+ T cells as opposed to CD4+ T cells within lesions. Furthermore, production of cytokines and the cytotoxicity levels of cells were diminished (212). The infiltration of regulatory T cells, which are known to be potent suppressors of inflammatory immune responses, was observed to remain elevated, thereby facilitating the progression of the disease (213, 214). Endometriosis has been infrequently identified in the lower genitourinary tract of males, primarily documented through case reports (215–217), with notable exceptions in transgender men (218). The predominant risk factor associated with male endometriosis, as suggested in previous cases, is prolonged exposure to estrogen therapy. However, there is a lack of published data regarding the microbiome and immune responses in male patients with this condition.

#### Polycystic ovary syndrome

PPCOS is one of the most prevalent endocrine disorders affecting women of reproductive age, often resulting in complications related to pregnancy and infertility. This condition is characterized by the excessive production of male sex hormones, known as androgens, by the ovaries. A notable feature of PCOS is chronic low-grade inflammation, which is associated with immune and metabolic disturbances, including obesity and insulin resistance. Numerous studies have demonstrated a significant correlation between PCOS and the composition of gut microbiota. Research has demonstrated that the abundance of two genera from *Clostridiales*, specifically *Ruminococcaceae* UCG-002, and the *Clostridiales* Family XIII AD3011 group, is correlated with various markers associated with PCOS in women. Furthermore, it has been identified that bacteria capable of producing gamma-aminobutyric acid, such as *Parabacteroides distasonis* and *Bacteroides fragilis*, are found in increased levels in PCOS-positive women (219). Additionally, the abundance of *Bacteroides vulgatus* was found to be increased gut microbiota of individuals diagnosed with PCOS (220).

Women diagnosed with PCOS frequently exhibit obesity and insulin resistance, which are pathophysiological conditions characterized by the transformation of macrophages from an anti-inflammatory M2 phenotype to a pro-inflammatory M1 phenotype, accompanied by increased secretion of interleukin-6 and intercellular adhesion molecule-1 (*ICAM-1*) (194, 221, 222). Furthermore, PCOS is associated with a significant reduction in activated and memory T lymphocytes within the theca layer of ovarian follicles (223).

It has been suggested that the primary defect of PCOS extends beyond an ovarian disorder to encompass a more comprehensive endocrine and metabolic disturbance that can affect men as well. The male phenotype associated with PCOS has been identified, although its characteristics remain ambiguous. In this regard, research indicates that male relatives of women diagnosed with PCOS may serve as male counterparts to the condition. This observation may indicate the presence of a genetic factor that drives PCOS, suggesting that PCOS is not solely a primary disorder of female reproduction. Instead, it may be conceptualized as a condition characterized by cardiometabolic dysregulation and hyperandrogenism, with ovarian dysfunction emerging as a secondary consequence (224, 225). Several potential PCOS phenotypes have been proposed for men, including abnormalities in male hair distribution, such as premature balding, as well as metabolic irregularities, including insulin resistance (224, 226).

In summary, we have described two reproductive disorders commonly observed in women that are mediated by the gut microbiome. In the context of male reproductive organs, there exists a connection between the gut microbiome and the male urogenital tract. The gut-testes axis refers to the interaction between the gut microbiome and its influence on the regulation of testicular function, which might play a significant role in male reproductive health and infertility. However, the vast majority of studies have been performed using animal models and were not discussed in this review.

### Gut-obesity axis

Obesity is a multifaceted metabolic disorder characterized by an unclear etiology, which is likely influenced by a combination of genetic and environmental factors. There exists conflicting evidence regarding the prevalence of obesity across genders. Some studies indicate that obesity is marginally more prevalent among females. However, in comparison to males, females appear to be less susceptible to various metabolic disturbances and sequelae associated with the progression of obesity-related diseases (227, 228). A recent study indicated that the prevalence of obesity is greater among men than women; however, women exhibit a higher percentage of body fat compared to men (229). The gut microbiome is increasingly acknowledged for its significant role in obesity, with notable sex-based differences influencing its effects on metabolic health and fat distribution. Variations in the composition of gut microbiota between males and females result in divergent metabolic and immune responses, which ultimately impact the risk, type, and severity of obesity. Haro et al. sought to identify differential gut microbiota signatures associated with obesity, taking into account gender and body mass index. The study found that the abundance of the *Bacteroides* genus was lower in men than in women, irrespective of obesity levels. However, the prevalence of this genus diminished in men as body mass index increased; however, it remained stable in women under similar conditions. This observation suggests that microbial shifts are more pronounced in men during weight gain.

Furthermore, a greater prevalence of the genera *Methanobrevibacter* and *Veillonella*, as well as the species *Bacteroides plebeius* and *Coprococcus catus*, was detected in men. In contrast, abundance of *Bilophila* and *Bacteroides caccae* were found in women regardless of obesity correlation. However, when men and women were adjusted for their body mass index, a higher *Firmicutes*/*Bacteriodetes* ratio was observed in women (229). A distinctive viewpoint on the disparities in gut microbiota composition is offered by the investigation of gender differences among schizophrenia patients with central obesity. A study published in 2022 demonstrated that female patients with central obesity displayed a significantly lower diversity of gut microbiota in comparison to their normal-weight counterparts, whereas no notable differences were found among male patients. This decrease in diversity, particularly characterized by diminished levels of beneficial bacteria such as *Verrucomicrobia* and *Akkermansia*, may play a role in the elevated metabolic risk linked to central obesity in women (230). In contrast, the abundance of *Prevotella* and *Roseburia* was observed to be elevated in females with central obesity, indicating potential sex-specific mechanisms through which gut dysbiosis may heighten the risk of obesity. Consequently, the modification of gut microbiota composition through dietary interventions could represent a promising approach for obesity management. Building upon this concept, it has been demonstrated that a moderately high-protein (MHP) diet and a low-fat (LF) diet yield distinct effects in men and women, with no corresponding bacterial changes observed between the sexes. Men subjected to the MHP diet exhibited a significant reduction in the abundance of *Negativicutes*, *Selenomonadales*, and *Dielma*. In contrast, women showed an increase in *Pasteurellales*, *Phascolarctobacterium succinatutens* and *Ruthenibacterium lactatiformans*. Furthermore, men on the LF diet experienced a notable increase in *Bacilli*, *Lactobacillales*, *Christensenellaceae*, *Peptococcaceae*, and *Streptococcaceae*, including *Peptococcus*, *Streptococcus*, and *Christensenella*, as well as *Duncaniella dubosii*. Conversely, women on the same diet exhibited a significant decrease in *Bacteroides clarus* and *Erysipelothrix inopinata* (231).

The relationship between adiposity and immune function is complex. Research indicates that, in general, eutrophic females exhibit a lower level of inflammation compared to eutrophic males; however, both obese females and males exhibited a heightened inflammatory response when compared to their eutrophic counterparts. A significantly higher total count of peripheral erythrocytes was observed in males, regardless of whether they belonged to the eutrophic or obese groups. Furthermore, eutrophic males exhibited a greater total lymphocyte count compared to eutrophic females. Additionally, a distinct inflammatory response was observed in relation to the secreted factors; males demonstrated elevated levels of TNFβ, IL15, and IL2, alongside reduced levels of IL10 and IL13. Conversely, obese females displayed increased concentrations of TNFα, CCL3, CCL4, and IP10 in the circulation (232).

In conclusion, sex-based differences in gut microbiota play a significant role in influencing the risk of obesity, fat distribution, and overall metabolic health. Women typically possess a gut microbiome profile that favors anti-inflammatory immune pathways and promotes healthier fat distribution. In contrast, men are more prone to exhibit gut microbial patterns that are associated with increased inflammation and heightened metabolic risk.

## Sexual dimorphisms in gut microbiome in centenarians

The gut microbiome has been identified as a potential contributor to the development of a favorable health phenotype that facilitates extreme longevity, as observed in centenarians. This section aims to elucidate the most recent evidence that positions the gut microbiome as a potential protective factor in the pursuit of extreme longevity, particularly through its association with centenarians. Several studies have examined the microbiome of centenarians from various geographic regions, including South Korea (233), Italy (234, 235), Japan (236), and China (237, 238). However, it is well established that factors such as ethnicity, habitat, and lifestyle significantly influence the gut microbiome, which complicates the ability to draw universal conclusions. In 2024, Chen et al conducted a comprehensive fecal metagenomic analysis involving eight long-lived populations from three countries: Japan, China, and Italy. The objective of this study was to investigate the associations between microbial composition and longevity-related traits in a large cohort. This approach will facilitate the identification of lifespan-associated microbiomes that are consistent across various ethnic populations from several geographic locations. The analysis revealed that several species were significantly enriched in the microbiota of long-lived individuals, including nonagenarians and centenarians, across multiple cohorts, namely *Eisenbergiella tayi*, *Methanobrevibacter smithii*, *Hungatella hathewayi*, and *Desulfovibrio fairfieldensis*. Further analysis revealed that the identified populations are engaged in various functional processes. *E. tayi* is involved in protein N-glycosylation, while *D. fairfieldensis* and *M. smithii* contribute to the biosynthesis of vitamin K2 and 3-dehydroquinate/chorismate, respectively. Additionally, *H. hathewayi* plays a role in the purine nucleobase degradation. Despite the conduct of a well-designed descriptive study, the focus on sex-specific aspects was overlooked (239).

In the same year, Luan et al. sought to elucidate the influences and contributions of sex on the gut microbiome in the context of healthy aging, using a cohort of healthy centenarians from Hainan. Their findings indicated that the composition and functional patterns of the microbiome exhibited significant variations between sexes, with 31 species identified as being enriched in males and 7 species enriched in females (240). Among species with a predominance of males, three probiotic *Lactobacillus* species have been identified. It is noteworthy that a higher abundance of certain bacteria in men’s gut microbiome, including *Eggerthella lenta*, *Clostridium hathewayi*, *Clostridium difficile*, *Anaerotruncus colihominis*, and *Actinomyces viscosus*, has been linked to unhealthy aging. In contrast, species associated with healthy aging were predominantly identified in female centenarians, including *Prevotella copri*, *Eubacterium rectale*, *Prevotella stercorea*, *Roseburia inulinivorans*, and *Coprococcus comes*. The study identified exceptions in the enrichment of *Dorea longicatena*, which has been associated with poorer cognitive performance (241), as well as *Lachnospiraceae bacteria*, which exhibit both beneficial and detrimental effects. On one hand, these bacteria are characterized as commercial probiotics; on the other hand, they are known to provide immunity-stimulating and homeostasis-maintaining effects (242). These findings suggest a significant difference in microbiome composition and functionality between sexes, which may speculatively contribute to the observed gender longevity gap.

## Discussion and conclusions

The consideration of gender in human biomedical research, particularly in the contexts of immunity and aging, is gradually gaining prominence. Although the majority of the reviewed studies are descriptive, correlative, and associative in nature, our understanding of sexual dimorphism has become clearer on one hand, while simultaneously revealing additional factors that remain inadequately explored and warrant further investigation. For example, males exhibit greater susceptibility to infections from birth through adulthood, suggesting that sex chromosomes play a significant role in the sexual dimorphism observed in innate immunity. However, the molecular mechanisms that modulate adaptive immunity and contribute to the increased vulnerability of males remain largely unknown. The sex-based regulation of immune responses ultimately influences the development of age-related diseases and life expectancy. A more comprehensive understanding of these mechanisms could facilitate the development of improved diagnostic and therapeutic strategies, thereby positively influencing healthy aging and longevity.

The aging process is characterized by alterations in immune system function. Given that the immune systems of young men and women exhibit significant differences, they experience distinct age-related immunological changes and aging consequences in a sex-specific manner. These sex-specific alterations in immunity during aging may lead to varying predispositions to health disorders and differing therapeutic outcomes. Therefore, sexual dimorphism should be regarded as a critical variable in the prophylactic and therapeutic management of age-associated diseases. Furthermore, sexual dimorphism also influences immune responses to various occupational activities, lifestyle choices, and individual characteristics, potentially affecting healthy aging and longevity. From this perspective, women appear to be more susceptible and may experience more severe responses to environmental factors.

The gut microbiome has emerged as a potential factor associated with the establishment of a favorable health phenotype, exhibiting a significant gender-specific influence. However, the mechanisms by which the gut microbiome contributes to conditions conducive to successful aging remain unclear. Consequently, future research should prioritize investigating the causal relationship between sexually dimorphic immunity and the microbiota while taking into account the aforementioned confounding factors.

It is evident that men and women pursue distinct trajectories in their quest for extreme longevity. The primary objective of the field of longevity research is not only to extend lifespan but also to promote a healthy life characterized by an active, productive, and mentally positive lifestyle. Consequently, this research domain is becoming increasingly multifaceted and necessitates more specialized investigations that take gender differences into account. Notably, while over 80% of the world’s centenarians are female, male centenarians tend to exhibit better health and are less susceptible to environmental factors. There is numerous data regarding the impact of genetic factors on the likelihood of women reaching extreme lifespan limits compared to men; however, the significance of longevity-associated genetic factors is highly variable and contingent upon ethnicity. It is clear that our understanding of human extreme longevity is largely associative and requires further investigation, including larger human cohorts across various countries, while considering genetic, medical, and social dimensions.

In conclusion, there is a pressing need for additional clinical applications to address the existing gap in human data concerning sexual dimorphism, immunity, and healthy aging. This research should particularly focus on the integration of bioinformatics methodologies capable of effectively managing and analyzing the highly dynamic and complex omics datasets commonly encountered in clinical studies. Furthermore, the incorporation of sex-specific thresholds in the analysis should be consistently implemented to prevent the oversight of significant gender-related findings.
